# Bi-photon spectral correlation measurements from a silicon nanowire in the quantum and classical regimes

**DOI:** 10.1038/srep12557

**Published:** 2015-07-28

**Authors:** Iman Jizan, L. G. Helt, Chunle Xiong, Matthew J. Collins, Duk-Yong Choi, Chang Joon Chae, Marco Liscidini, M. J. Steel, Benjamin J. Eggleton, Alex S. Clark

**Affiliations:** 1 Centre for Ultrahigh bandwidth Devices for Optical Systems (CUDOS), Institute of Photonics and Optical Science (IPOS), School of Physics, University of Sydney, New South Wales 2006, Australia; 2 CUDOS and MQ Photonics Research Centre, Department of Physics and Astronomy, Macquarie University, New South Wales 2109, Australia; 3 Dipartimento di Fisica, Universita degli Studi di Pavia, via Bassi 6, I-27100 Pavia, Italy; 4 Laser Physics Centre, Australian National University, Canberra, Australian Capital Territory 2913, Australia; 5 NICTA-VRL, The University of Melbourne, Victoria 3010, Australia (now with Advanced Photonics Research Institute, GIST, Korea)

## Abstract

The growing requirement for photon pairs with specific spectral correlations in quantum optics experiments has created a demand for fast, high resolution and accurate source characterisation. A promising tool for such characterisation uses classical stimulated processes, in which an additional seed laser stimulates photon generation yielding much higher count rates, as recently demonstrated for a *χ*^(2)^ integrated source in A. Eckstein *et al.* Laser Photon. Rev. **8**, L76 (2014). In this work we extend these results to *χ*^(3)^ integrated sources, directly measuring for the first time the relation between spectral correlation measurements via stimulated and spontaneous four wave mixing in an integrated optical waveguide, a silicon nanowire. We directly confirm the speed-up due to higher count rates and demonstrate that this allows additional resolution to be gained when compared to traditional coincidence measurements without any increase in measurement time. As the pump pulse duration can influence the degree of spectral correlation, all of our measurements are taken for two different pump pulse widths. This allows us to confirm that the classical stimulated process correctly captures the degree of spectral correlation regardless of pump pulse duration, and cements its place as an essential characterisation method for the development of future quantum integrated devices.

In the last decade the investigation of non-classical correlations between photons has been one of the central topics in quantum optics. Quantum entanglement between photon pairs is a key resource for exceeding the technological limits imposed by classical physics and plays an integral part in many applications of quantum optics including optical quantum computing^1^, secure communication over large distances^2^,^3^ and quantum metrology^4^. The need for complex and precisely controlled correlated photon states is the driving force behind the development of new methods to accurately characterise correlated photon pair sources.

Quantum entanglement between photons can exist in many degrees of freedom including polarisation, time-bin, and energy. A common form of entanglement arises from energy-time correlation, known as spectral entanglement, which is particularly important in quantum communications^5^,^6^,^7^,^8^. Spectrally entangled photons arise naturally^9^ in spontaneous parametric down-conversion (SPDC) and spontaneous four-wave mixing (SFWM), in second order (*χ*^(2)^) and third order (*χ*^(3)^) nonlinear materials respectively, as a result of the ultrafast nonlinear interaction and energy-matching requirements. Until very recently, the most common method for characterising the degree of spectral correlation of photon pairs has been the direct measurement of the joint spectral intensity (JSI). This function, defined formally below, is essentially the probability distribution in frequency space for detecting pairs. Generally, the JSI is obtained by performing photon coincidence measurements in which the correlated photon pairs are detected via a pair of single photon detectors over a range of frequencies (see Fig. 1). This has been performed a number of times for SPDC using a tunable filter^10^ or a highly dispersive fibre^11^, and for SFWM using either temporal dispersion in long lengths of fibre^12^, monochromators^12^,^13^, or spatial mode separation of the signal and idler photons^14^. All of these schemes suffer from limitations in both achievable resolution and acquisition times. The latter are typically large due to the need to measure sufficient coincidences to obtain a satisfactory signal to noise ratio, usually in the presence of low throughput. Clearly, rapid high-resolution characterisation of the spectral correlations created in nonlinear pair sources is challenging.

To address this problem, Liscidini and Sipe^15^ introduced a technique to reconstruct the JSI by performing stimulated nonlinear wave mixing. This approach uses bright classical fields, exploiting the observation that, for a given pumping scheme and nonlinear device, the spontaneous and simulated frequency conversion response functions can be made mathematically identical. This was demonstrated recently in a *χ*^(2)^ device, namely an AlGaAs ridge waveguide^16^, where the spontaneous process of SPDC was compared to the stimulated process of difference frequency generation (DFG). The experiment compared the JSIs obtained from SPDC via a temporal dispersion method and stimulated DFG via an optical spectrum analyser (OSA). The stimulated process using DFG produced a higher resolution in only a third of the collection time. There have also been two demonstrations of reconstruction of the JSI via stimulated four-wave mixing (FWM) in a *χ*^(3)^ nonlinear device, namely a birefringent optical fibre^17^ and a silicon ring-resonator based photonic chip^18^. The first work compared the JSI obtained from the stimulated process to that taken by coincidence measurements on a similar, though not identical fibre finding close resemblance. The second work compared the JSI obtained from the stimulated process to a theoretical model, finding good agreement between the two.

Here we apply the stimulated process concept to an integrated *χ*^(3)^ nonlinear device, in this case a silicon nanowire, and compare the results to quantum measurements for two different pump pulse widths. Silicon photonics is currently a leading platform for on-chip quantum integrated circuits^19^,^20^, due to the high intrinsic *χ*^(3)^ nonlinearity, the possibility for dense integration, mature fabrication methods, low losses and low cost^21^. As such, there is significant motivation to use integrated *χ*^(3)^ nonlinear devices for generating quantum correlated photon pairs in the telecommunications ban^22^,^23^,^24^,^25^,^26^,^27^,^28^,^29^ and to develop fast characterisation techniques. In the following, we measure three JSIs, one via coincidence measurements from SFWM, and two via stimulated FWM using different detection methods. The classical stimulated FWM techniques produce fast and reliable results, which can be readily extended to larger frequency ranges and are directly applicable to many future integrated nonlinear devices. Moreover, we observe and compare the change in the spectral correlation of photon pairs generated using two different pump pulse durations in the nonlinear device.

**Formalism** To understand the relationship between SFWM and stimulated FWM, we refer to Fig.1, which illustrates the annihilation of two pump photons resulting in the generation of a signal and idler photon of higher and lower energy, respectively. For both processes, the frequencies must obey energy conservation such that
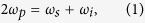
where *ω*_*p*_, *ω*_*s*_ and *ω*_*i*_ are the pump, signal and idler frequencies respectively. SFWM occurs in the absence of any seed field, and instead relies on vacuum fluctuations to seed the conversion of a pair of pump photons into correlated signal and idler photons. In contrast, stimulated FWM involves a classical seed field in either the signal or idler band and is much more efficient. It forms the basis for parametric oscillators^30^,^31^ and ultra-broadband amplifiers^32^.

For guided-mode co-polarised photon generation via SFWM, the two-photon component of the output squeezed state can be expressed as
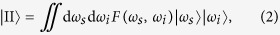
where 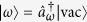 is the state containing a single photon in the waveguide mode at *ω*. Note that the two emitted photons occupy the same waveguide mode. Additionally,
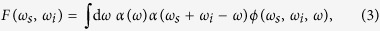
is known as the bi-photon wavefunction or joint spectral amplitude (JSA). Its squared modulus 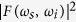 defines the JSI. The function *α*(*ω*) is the complex amplitude of the pump spectrum (with centre frequency *ω*_*p*_) and *ϕ*(*ω*_*s*_, *ω*_*i*_, *ω*) is the phase-matching function of the waveguide which reflects the waveguide material and design properties. As a complex function of two variables, the JSA can be usefully analysed in terms of the Schmidt decomposition, by which it is expressed as a linear combination
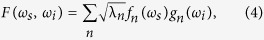
where *f*_*n*_(*ω*_*s*_) and *g*_*n*_(*ω*_*i*_) are each a complete set of orthonormal functions, and *λ*_*n*_ are positive real numbers known as the Schmidt magnitudes satisfying 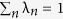. This can then be used to quantify the degree of entanglement in the system via the Schmidt number 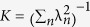
33. For a completely uncorrelated system the Schmidt magnitudes are λ_*n*=1_ = 1 and λ_*n*≠1_ = 0 so that *K* = 1. However for a correlated system, multiple Schmidt magnitudes are nonzero so that *K* > 1 (see Fig. 1: JSI plot inset).

In fact, obtaining the full phase-dependent JSA for a bi-photon source is experimentally challenging^34^, and experiments to date have focused on measuring the JSI represented by 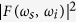, as we do here. Unfortunately, this JSI measurement results in a loss of phase information when estimating 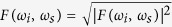. Additionally, the Schmidt decomposition is not directly applicable to 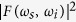, and so the Schmidt number *K* is not strictly available from experiment. However, a singular value decomposition (the matrix analog of the Schmidt decomposition,) applied to the square root of the measured JSI, 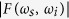, does give a lower bound to the Schmidt number^16^, which remains a useful characterisation of the source. In the following, we refer to the Schmidt number lower bound (SNLB), with the symbol 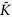.

## Experimental Methods

In this work we demonstrate three distinct methods of obtaining JSIs from a *χ*^(3)^ nonlinear device using quantum, singles-based and OSA measurements that provide progressive improvements to the signal-to-noise ratio and measurement efficiency. We first employ a high resolution spatial separation method^14^ to determine the JSI in the quantum regime by measuring the correlated photon pair coincidences from SFWM. In the second experiment, we employ an additional narrow-band seed laser tuned across the signal band to stimulate classical FWM, and measure the spectrum of the generated idler field using a single photon detector. We refer to this as the singles-based approach. Our final method again involves the measurement of the idler field generated via stimulated FWM, but in this case using a high resolution optical spectrum analyser (OSA). We refer to this as the OSA method. We compare our experimental methods for two different laser pump pulses and thus observe a change in the spectral correlations of the photon pairs generated in our nonlinear device.

The experimental setup is shown in Fig. 2. It consists of three major parts required to perform SFWM coincidence measurements and traditional stimulated FWM measurements in the nonlinear device: the pump and seed laser preparation, the nonlinear device, and the detection and analysis setup.

### Pump and Seed Preparation

Figure 2(a) shows the different pump and seed laser preparations. The first pump source was a pulsed fibre laser (Pritel) centred at 1550 nm (Fig. 2(a)(i)) which produced 10 ps pulses with a repetition rate of 50 MHz and a 70.8 GHz spectral full width at half maximum (FWHM). The pulses passed through a polarisation controller (PC) to select TE polarisation with respect to the waveguide device, an isolator (ISO) to protect the laser and a variable attenuator (ATT) to tune the input pump power. Residual cavity photons from the laser were removed using a narrowband tunable band-pass filter (TBPF) before entering a 99:1% coupler to monitor the input power entering the nonlinear device on a power meter (PM).

The second pump source, shown in Fig. 2(a)(ii), used one channel of an external cavity diode laser (ECDL) centred at 1550 nm which passed through a PC before being pre-amplified by a low-noise erbium doped fibre amplifier (EDFA) to directly increase the pump signal. The pump wave was modulated to 270 ps Gaussian pulses at a repetition rate of 100 MHz by a lithium niobate intensity modulator (IM, Sumitomo) driven by a pulse generator (PG, AVTech), resulting in a 10.4 GHz spectral FWHM. The pump pulse stream was then amplified by a second EDFA and subsequently filtered by two arrayed waveguide gratings (AWGs, JDSU) to remove any amplified spontaneous emission noise. A PC was placed in between the two AWGs to adjust the polarisation such that the pump pulse was TE polarised in the nonlinear device.

Finally, the seed laser for the stimulated FWM experiments, shown in Fig. 2(a)(iii), used the second channel of the ECDL which was also set to TE polarisation using a PC. This channel of the ECDL was computer controlled to repeatedly scan the higher-band channel over the desired spectral detuning range from the pump, detailed below.

### Nonlinear Device

As sketched in Fig. 2(b), our nonlinear device is a 3 mm long silicon-on-insulator (SOI), 220 nm high by 460 nm wide buried silicon nanowire (SiNW), providing an effective nonlinearity of approximately *γ* ≈ 236 W^−1^m^−1^. This *γ* was calculated via computer simulation of waveguide modes, and was used to estimate the largest average number of SFWM pairs generated on-chip per pulse within our filtering window that we could expect to observe. To ensure that we were always probing the two-photon component of our output state, we kept this number less than 0.1 in all measurements. In particular, for our brightest measurement, using the *τ* = 10 ps (*R*_rep_ = 50 MHz) laser at a detuning of Ω = 0.8 THz from the pump, for a Δ*v* = 10 GHz filtering, 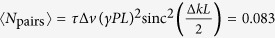 per pulse. Here the peak power 
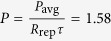
 W, nonlinear device length *L* = 0.003 m and phase mismatch Δ*k* = 729.0 m^−1^. This value is corroborated by a measured pair collection rate of 
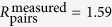
 per second when accounting for approximately *α* = 31.9 dB of losses per channel including propagation and coupling losses, which we compare with 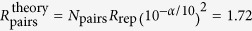 per second, demonstrating good agreement between theory and experiment. We note that this high peak power does have a modest effect on the phase matching condition Δ*k* = *β*_2_(2*π*Ω)^2^ + 2*γP*, but that any quantum time-ordering effects are expected to be quite small as we are not operating in a nearly frequency uncorrelated regime^35^.

To improve waveguide to fibre coupling efficiency, the TE-optimised waveguide was inverse tapered over a 200 *μ*m length to a cross section of 220 nm high by 130 nm wide at the facet. The SiNW was fabricated from a SOI wafer using standard photolithography and reactive ion etching, followed by the addition of a 2 *μ*m silicon dioxide upper-cladding layer deposited via plasma-enhanced chemical vapour deposition. The average power in front of the waveguide was 4.9 mW and 790 *μ*W for the 270 ps (100 MHz) and 10 ps (50 MHz) lasers respectively. These powers were set to generate the same number of photon pairs per second in the device for the two laser pulse widths and were below the threshold for two-photon absorption^36^. The average seed power in front of the waveguide was kept constant at 36.5 *μ*W. The TE propagation and coupling losses of the SiNW were approximately 2–2.5 dB/cm and 2–2.5 dB/facet respectively. The total loss between the input and output of the SiNW was approximately 4.5 dB for all measurements.

### Detection and Analysis

Three different experimental setups were used for the detection and analysis of the photon pairs generated by SFWM and the photons generated by stimulated FWM. The first setup, shown in Fig. 2(c)(i), was used to measure the quantum correlations from SFWM by coincidence detection. A 99:1% coupler was used to monitor the 1% output power exiting the SiNW via a PM. The remaining 99% was sent to a multi-output liquid-crystal-on-silicon waveshaper (LCoSWS, Finisar Waveshaper) that separated idler and signal photons into distinct spatial mode channels. The two channels were then broadband filtered to remove any residual pump photons before entering another two PCs inserted before the two superconducting single photon detectors (SSPDs, Single Quantum) to optimise the detection efficiency of the two channels. Coincidence measurements were conducted and recorded by a computer via a time interval analyser (TIA, SensL). The spectral resolution obtained in each channel was 10 GHz, limited by the pixel bandwidth of the LCoSWS. This led to a 40 × 40 pixel grid for the final JSI.

The next setup, shown in Fig. 2(c)(ii), implemented the singles-based characterisation of the JSI. In addition to the pump pulse, to stimulate FWM the seed laser described in Fig. 2(a)(iii) was also injected into the SiNW at a higher frequency than the pump, corresponding to the measured signal band in SFWM measurements. The generated average power in the idler band was approximately 1.8 *μ*W. Instead of performing coincidence measurements, we measured the singles count rate recorded by one SSPD in the idler detection band, with the seed laser operating in the signal band. Both the seed laser frequency and the idler detection band (controlled by the LCoSWS) were scanned in 10 GHz units in a raster scanning fashion. Again, the spectral resolution obtained was 10 GHz with the extracted JSI represented on a 40 × 40 pixel grid. A 20 dB attenuation was applied in the LCoSWS to limit the rate of idler photons being detected by the SSPD, thus avoiding saturation.

The final measurement setup, shown in Fig. 2(c)(iii), is the OSA measurement of the JSI using stimulated FWM. In this measurement we kept the scanning seed laser as in the singles-based measurement, but replaced the LCoSWS, PC, SSPD and TIA with an optical spectrum analyser (OSA, Yenista) that provided a higher resolution of 2.5 GHz. The resulting JSI has 16 times higher resolution with a 157 × 157 grid.

### Theoretical calculations

To theoretically model the JSA for the different laser pulses, the SiNW dispersion relation was approximated as
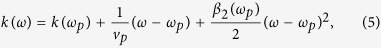
where *k*(*ω*_*p*_) = 9.63 × 10^6^ m^−1^, *v*_*p*_ = 7.02 × 10^7^ m/s and *β*_2_(*ω*_*p*_) = −6.03 × 10^−25^ s^2^/m. Using these parameters and the geometry of the waveguide, we used Eq. (3) to calculate the expected JSA and JSI for the two laser pulses. The resulting JSI distributions are shown in Fig. 3(a)(i). As expected, for pulses increasing in duration towards quasi-CW, the high SNLB in Fig. 3(a)(i) indicates a more highly spectrally correlated state compared with Fig. 3(b)(i). This regime of very strong frequency correlations is particularly important in quantum information applications as many protocols rely on time-frequency entanglement for use in, for example, quantum cryptography^3^ and quantum computation^1^.

### Limitations

**Figure 1:**
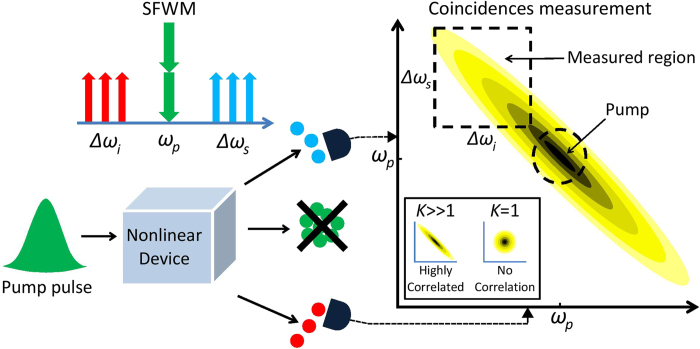
Schematic illustration of the SFWM process and a conventional JSI measurement. A pulse is injected into a nonlinear device generating a signal and idler photon via the annihilation of two pump photons. The unconverted excess pump photons are dropped. Measuring correlations across two detectors, the JSI is obtained by recording the number of coincidences obtained at each specific idler and signal frequency.

Unlike some SPDC and SFWM schemes that are phase-matched far from the pump, our SiNW dispersion does not allow for measurement of the whole JSI as the pump frequency lies in the centre (see Fig. 1 JSI plot). However this is not a serious restriction, since this band will also be inaccessible in any application of such a source. Our measurement is therefore concerned with an experimentally accessible portion of the JSI, over a tuning range of 0.745–1.135 THz (5.94–9.15 nm) from the centre frequency of the pump. The complete theoretical JSI was calculated and SVD was performed on that part of the spectrum accessible to our measurements. We then used the SNLB associated with this experimentally accessible portion to quantify the accuracy of each of our measurements. However, the measured values of 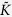 are affected by the available frequency resolution, as well as the noise in each class of measurement. To understand the impact of limited resolution, and thus separate this from the impact of noise in the experimental data, for each of the pump pulse lengths, we calculated the expected theoretical values of 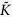, at each of the available frequency resolutions and a reference value at much finer resolution beyond which 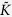 does not change in the 4th decimal place. Note that in our case, the combination of accessible frequency range and dispersion strength meant that no difference was found between the value of the SNLB, 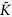, and the true Schmidt number, *K*, in the high resolution calculations. This would not be true in general of course.

The expected impact of limited resolution is shown in Table 1. It is clear that for the narrow bandwidth 270 ps source, the maximum observable value of 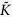 is significantly reduced from its ideal value. On the other hand, for the broadband 10 ps source, even the coarse 40 × 40 grid can represent a 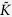 exceeding 80% of the ideal value. The extent to which the measured values fall below these limits is a measure of the impact of noise of various types.

## Results

With the combination of the two laser pulses and the three detection methods, we measured a total of six partial JSIs. The theoretical and the three experimental JSI measurements are shown in Fig. 3(a) for the 270 ps and 10 ps pulses respectively, with their associated SNLBs 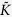 estimated by singular value decomposition and their respective errors estimated from Monte Carlo simulations. These simulations re-sampled each point of each measured JSI from a Poissonian distribution to create 10^5^ new Poissonian distributed JSIs for each measured JSI. Then the SNLB was calculated for each of the 10^5^ new JSIs for each measurement, and the standard deviation of the distribution of the SNLBs were then used as the error in the SNLBs. Note the slight curvature and decrease in brightness of the curves from top left to bottom right in the ideal modelled JSIs in Fig. 3(a)(i). As the JSI is a convolution of the pump profile and the phase matching function (recall Eq. (3)), we would expect the JSI to decay to background if we continued to measure it beyond 1.135 THz from the central frequency of the pump.

The total time taken to build up the 40 by 40 pixel grid (10 GHz resolution) coincidence JSI plots shown in Fig. 3(a)(ii) was approximately 36 hrs and 33 hrs respectively. During this time we continually adjusted the LCoSWS pass band for each channel across the whole JSI at a rate of 6 pixels per minute, summing the pixels from each scan until the largest number of recorded coincidence counts in any one pixel was 105 with a Poissonian error of 10. This repeated sampling process was designed to minimise the effect of slow fluctuations in laser power and waveguide couplings. Using this method for coincidence measurements, the high integration times can also be attributed to the LCoSWS rejecting most of the generated signal and idler photons that are not captured by pass band. As theoretically predicted, the broader spectral profile *α*(*ω*) of the 10 ps laser source results in a broader anti-diagonal band, and thus a lower SNLB, for its associated JSI than that associated with the 270 ps source. As this is a SFWM measurement, the impact of accidental coincidences in JSI plots is large and contributes to a lower extracted SNLB than predicted.

**Figure 2:**
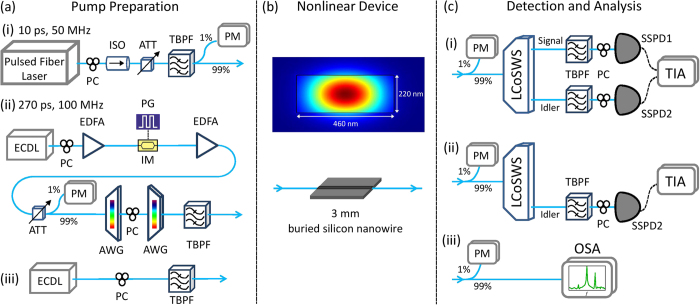
Schematic of all fibre-based JSI measurement setups showing (**a**) the pulsed fibre laser, polarisation controller (PC), optical isolator (ISO), variable attenuator (ATT), tunable band-pass filter (TBPF), power meter (PM), external cavity diode laser (ECDL), erbium-doped fibre amplifier (EDFA), pulse generator (PG), intensity modulator (IM), arrayed waveguide grating (AWG), (**b**) buried silicon nanowire (SiNW) and the simulated 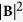 energy density of the fundamental TE mode, (**c**) liquid-crystal-on-silicon dynamically tunable filter (LCoSWS, Finisar WaveShaper), superconducting single photon detector (SSPD, Single Quantum—polarisation sensitive), time interval analyser (TIA) and optical spectrum analyser (OSA).

**Figure 3:**
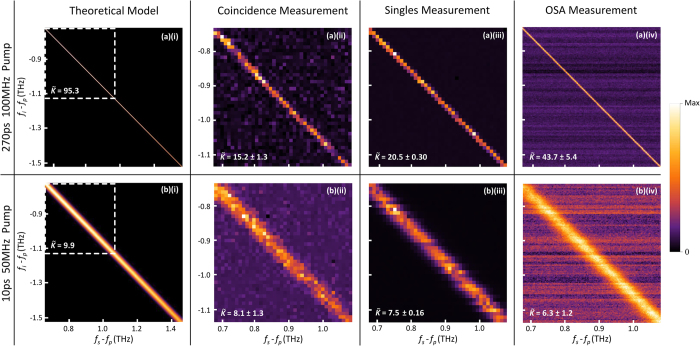
The theoretically-calculated model and results of the six JSI measurements, for (**a**) the 270 ps and (**b**) 10 ps pump laser pulses: (**i**) scaled theoretical ideal model with dashed box representing the measured region, (**ii**) photon pair coincidence measurement, (**iii**) stimulated FWM singles-based measurement and (**iv**) stimulated FWM OSA measurement. The brightest pixel in each plot (Max of the colour bar) corresponds to: (**a**)(**ii**) 105 coincidences, (**b**)(**ii**) 105 coincidences, (**a**)(**iii**) 318,720 counts, (**b**)(**iii**) 182,210 counts, (**a**)(**iv**) 1716.7 nW/10 GHz, and (**b**)(**iv**) 464.1 nW/10 GHz. These maxima all have Poissonian error except those for (**a**)(**iv**) and (**b**)(**iv**) which have an inherent error from the OSA. SNLBs are shown for each plot with errors calculated from Monte Carlo simulations.

The generated single photon measurements corresponding to the experimental setup in Fig. 2(c)(ii) are plotted in Fig. 3(a)(iii). As stimulated FWM leads to a count rate at a single detector on the order of 10^5^ s^−1^, very low relative numbers of background singles are seen when scanning the LCoSWS band pass filter. The counts in the dark background region are only limited by dark counts from our detectors, which are on the order of 100 s^−1^.

In this case, a higher signal-to-noise ratio in turn results in a higher SNLB being obtained when compared with the coincidence measurements, evident in the 270 ps pumped singles measurement in Fig. 3(a)(iii). However, a slightly lower SNLB was obtained for the 10 ps laser pulse in Fig. 3(b)(iii) compared with the corresponding coincidence measurement. This is caused by the non-uniform distribution of singles across the anti-diagonal band of the plot which is a result of small fluctuations in the laser powers, detector efficiency, and polarisation from scan to scan. Additionally, the 10 ps pumped coincidence value for the 40 × 40 grid (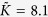) provided the closest agreement to the expected value (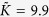) when compared with the singles-based measurement. Due to the high rate of stimulated FWM idler photon generation, the integration time for both pump measurements was limited only by the scanning speed of the seed laser and the LCoSWS, as well as the speed of the electronic acquisition. Thus the fastest possible integration time for both measurements was only 1.5 hours. Still, moving to this singles-based measurement results in a significant decrease in the required integration time when compared to the coincidence measurement, while providing comparable SNLBs.

The classical OSA measurements shown in Fig. 3(a)(iv) were completed within 2 hours for each laser pulse width but with 16 times higher resolution, at the maximum resolution of 2.5 GHz. The horizontal streaks visible in both JSI plots are a result of the constant change in the noise floor of the OSA with every trace measurement. In theory, the streaks can be eliminated by averaging multiple traces for a fixed seed frequency, but not without increasing the total integration time of each JSI measurement. In principle, using this method we are able to measure the complete JSI profile of the SiNW, as the OSA is not saturated by the input pump at the powers used here.

### Conclusion and Outlook

We have presented measurements comparing JSIs from a *χ*^(3)^ nonlinear device, in our case a SiNW, via three different experimental methods that can be used to characterise the correlations between generated photon pairs. This is achieved by employing both quantum correlation measurements and classical stimulated measurements, which makes use of the relationship between SFWM and stimulated FWM. For the stimulated FWM processes, we have shown two techniques, one that uses no further components than quantum correlations, other than a CW probe laser, and the other using a high resolution OSA. By successfully measuring the JSI in the quantum and classical regimes for two different laser pulses, we observed a direct change in the spectral correlation of the generated photon states, proving the versatility of our characterisation schemes. For the JSI measurements, we saw by switching from the quantum to classical measurements, we were able to increase the resolution from 10 GHz using the LCoSWS to 2.5 GHz using the OSA. However this also resulted in horizontal streaks in the JSI, a problem attributed to fluctuations in the noise floor of the OSA, and increased the relative error in the SNLB. In the future this could be overcome by using a lower noise OSA or limiting measurements to nonlinear devices with a higher FWM conversion efficiency as we would be operating further from the noise floor of the OSA. By comparing the SNLBs calculated via SVD of our quantum and classical measurements with our ideal theoretical model, we conclude that the OSA provided the most accurate spectral correlation measurement (although half of the predicted SNLB) for the long pump pulse. The measured spectral correlation for the short pump pulse via coincidence measurement provided us with the smallest deviation from the ideal model however, the measurement obtained via the OSA has a larger disagreement with the theory as a result of the horizontal streaks in the JSI measurement. Note that the stimulated FWM method provides the fastest measurement speed for a given resolution and thus the OSA measurement will consistently provide the highest resolution for future measurements of JSIs. Overall, the long pump pulse spectral correlation measurement provided us with the biggest discrepancy when compared to the ideal model, caused by the discretised JSI measurements having a limited resolution at the same scale as the pump spectral profile. Although not possible with the LCoSWS at this time, in theory the measurement resolution could be improved by reducing the LCoSWS programmable bandpass filter bandwidth. However, if we were able to reduce the LCoSWS filter bandwidth to the OSA resolution of 2.5 GHz for measurement in the coincidence counting regime, the measurement time would increase by a factor of 16, meaning a JSI measurement would take approximately 24 days of continuous measurements.

In the future these methods could be applied to other integrated pair generation devices including ring resonators^24^,^27^ and slow-light photonic crystals^25^,^26^,^28^,^29^. The methods presented here are of substantial importance for the characterisation of spectrally complex two photon states, particularly for nonlinear devices that require fast and reliable measurements, and when large numbers of devices must be characterised for use in future quantum technologies.

## Additional Information

**How to cite this article**: Jizan, I. *et al.* Bi-photon spectral correlation measurements from a silicon nanowire in the quantum and classical regimes. *Sci. Rep.* **5**, 12557; doi: 10.1038/srep12557 (2015).

## Figures and Tables

**Table 1:** Theoretically extracted SNLBs, 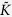 , for a 40 by 40, 157 by 157 and ideal JSIs, for the 270 ps 100 MHz and 10 ps 50 MHz laser pump pulses.

**Pump Pulse Duration**	**40 by 40**	**157 by 157**	**Ideal** 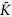
	**grid** 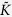	**grid** 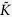	
270 ps	39.0	83.5	95.3
10 ps	8.1	8.4	9.9
